# Comprehensive pan-cancer analysis of YBX family reveals YBX2 as a potential biomarker in liver cancer

**DOI:** 10.3389/fimmu.2024.1382520

**Published:** 2024-04-18

**Authors:** Ze Yuan, Binbin Li, Wenmin Liao, Da Kang, Xinpei Deng, Hailin Tang, Jindong Xie, Dandan Hu, Aiqin Chen

**Affiliations:** ^1^ State Key Laboratory of Oncology in South China, Guangdong Provincial Clinical Research Center for Cancer, Sun Yat-Sen University Cancer Center, Guangzhou, China; ^2^ Department of Medical Oncology, The Third People’s Hospital of Yongzhou, Yongzhou, China

**Keywords:** pan-cancer, YBX family, tumor microenvironment, single-cell, liver cancer

## Abstract

**Background:**

The Y-box-binding proteins (YBX) act as a multifunctional role in tumor progression, metastasis, drug resistance by regulating the transcription and translation process. Nevertheless, their functions in a pan-cancer setting remain unclear.

**Methods:**

This study examined the clinical features expression, prognostic value, mutations, along with methylation patterns of three genes from the YBX family (YBX1, YBX2, and YBX3) in 28 different types of cancer. Data used for analysis were obtained from Cancer Genome Atlas (TCGA) and Genotype-Tissue Expression (GTEx) databases. A novel YBXs score was created using the ssGSEA algorithm for the single sample gene set enrichment analysis. Additionally, we explored the YBXs score’s association with the tumor microenvironment (TME), response to various treatments, and drug resistance.

**Results:**

Our analysis revealed that YBX family genes contribute to tumor progression and are indicative of prognosis in diverse cancer types. We determined that the YBXs score correlates significantly with numerous malignant pathways in pan-cancer. Moreover, this score is also linked with multiple immune-related characteristics. The YBXs score proved to be an effective predictor for the efficacy of a range of treatments in various cancers, particularly immunotherapy. To summarize, the involvement of YBX family genes is vital in pan-cancer and exhibits a significant association with TME. An elevated YBXs score indicates an immune-activated TME and responsiveness to diverse therapies, highlighting its potential as a biomarker in individuals with tumors. Finally, experimental validations were conducted to explore that YBX2 might be a potential biomarker in liver cancer.

**Conclusion:**

The creation of YBXs score in our study offered new insights into further studies. Besides, YBX2 was found as a potential therapeutic target, significantly contributing to the improvement of HCC diagnosis and treatment strategies.

## Introduction

Proteins in YBX family share an evolutionarily conserved cold-shock domain (CSD), acting a multifunctional role by controlling the regulation of transcription and translation (1–5). The YBX family participates in multiple cellular processes, such as controlling gene expression and protein synthesis, modifying RNA molecules, repairing DNA, packaging mRNA, and responding to external signals and stress (6–8). The oncogenic effects attributed to YBX identified to date stem from its transcription and translation functions of numerous genes implicated in cell growth, malignant transformation and drug resistance. In human, the YBX family 3 members, including YBX1, YBX2 and YBX3. YBX1, or YB-1, was initially identified in 1988 and is known for its specific binding to the CCAAT box and Y box. It plays a role as a negative regulatory factor through nuclear translocation (1). YBX proteins have also been reported to be associated with tumor proliferation, metastasis, invasion and drug resistance (9–14).

Numerous research studies have elucidated a correlation between the expression of YBXs and the progression and dissemination of malignant tumors. YBX1 is the first and most studied one among YBX family. The transcriptional regulation of YBX1 has been demonstrated to induce epithelial-mesenchymal transition (EMT) in various cancers (9, 15). Moreover, extensive research has presented compelling evidence highlighting the pivotal involvement of YBX1 in the progression, metastasis, and development of drug resistance across diverse cancer types, including lung squamous cell carcinoma, intrahepatic cholangiocarcinoma, hepatocellular carcinoma (HCC), renal cell carcinoma, and nasopharyngeal carcinoma (16–21). It has also been reported that YBX2 is correlated with the stemness, chemoresistance, malignancy and prognosis of endometrial cancer and lung cancer (22–26). Furthermore, YBX3 has been implicated in the progression of bladder and colon cancer (27, 28). In certain types of cancer, the involvement of YBXs in TME has also been demonstrated recently. The findings indicate that YBX genes potentially play roles specific to pancreatic adenocarcinoma in tumor progression (29). Consequently, the associations and mechanisms underlying the YBX family’s involvement in particular cancer types warrant further investigation. In recent years, an increasing number of studies have utilized bulk and single-cell techniques to explore the biological roles of target molecules in different cancer types, which have shown a certain level of efficacy (30, 31).

**Figure 1:**
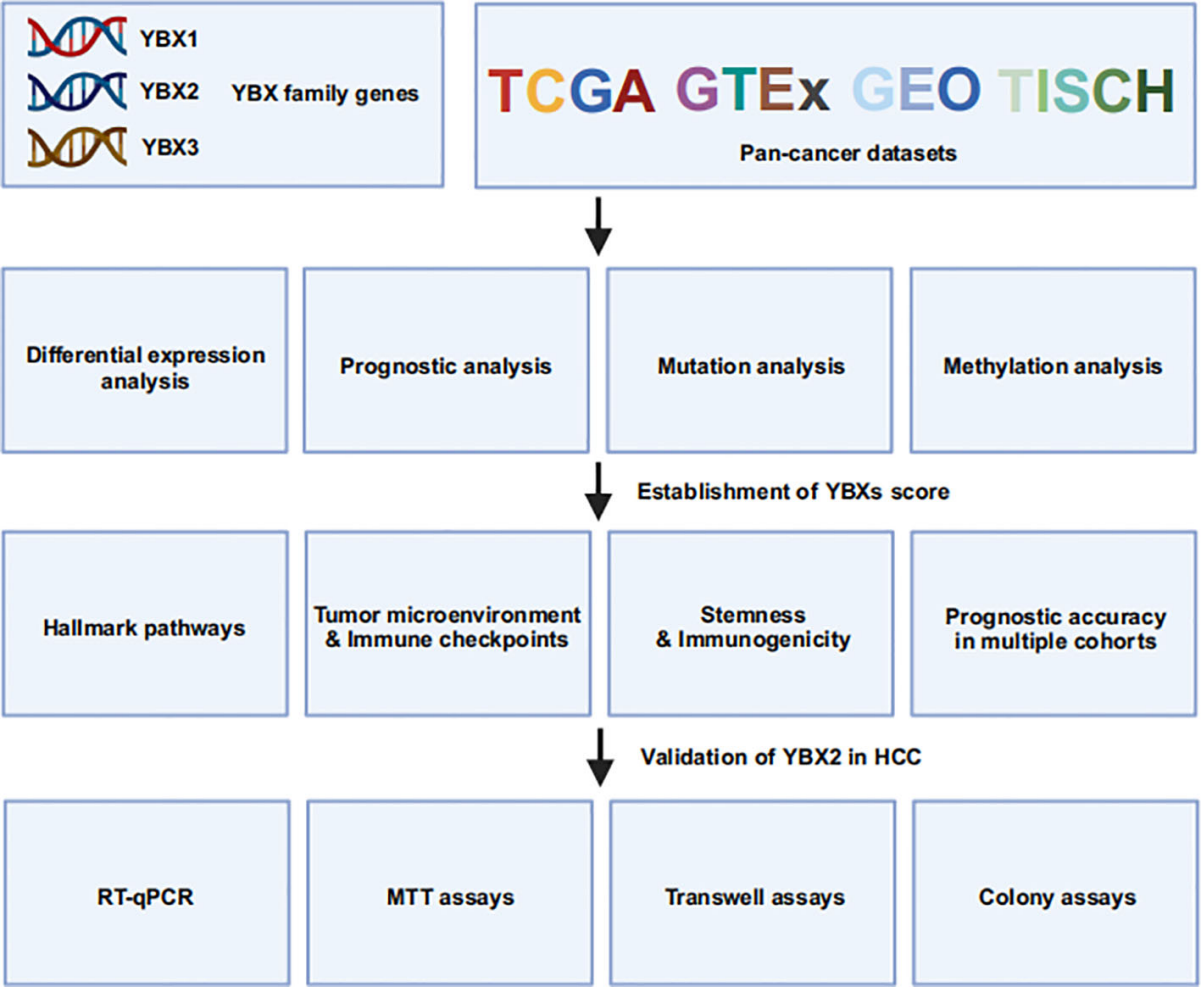
The flowchart of this study.

In this study, we conducted a comprehensive pan-cancer analysis of the YBX family genes, examining their expression, prognostic significance, mutations, methylation patterns, and clinical characteristics. TME, functioning as a conducive milieu for cancer cell proliferation, plays a crucial role in both tumor initiation and metastasis (32). Furthermore, the immune-activated tumor microenvironment, comprising immune cells that impede tumor development, is strongly connected to various treatment modalities such as chemotherapy, radiation therapy, targeted therapy, and immunotherapy (13, 33, 34). Based on this analysis, we developed a YBXs score, validated through a comprehensive analysis of multiple cohorts, and examined its correlation with the TME, clinical outcomes, and resistance to drugs. Finally, we validated the increased expression of YBX2 in some HCC cell lines and its correlation with enhanced HCC proliferation, migration, and invasion, thereby addressing our previous pan-cancer analysis findings. In summary, our findings offer a comprehensive pan-cancer analysis of the YBX family, potentially aiding in prognosis monitoring and the development of diverse therapeutic strategies (**Figure 1**).

## Results

### Expression level of YBX family genes

**Figure 2:**
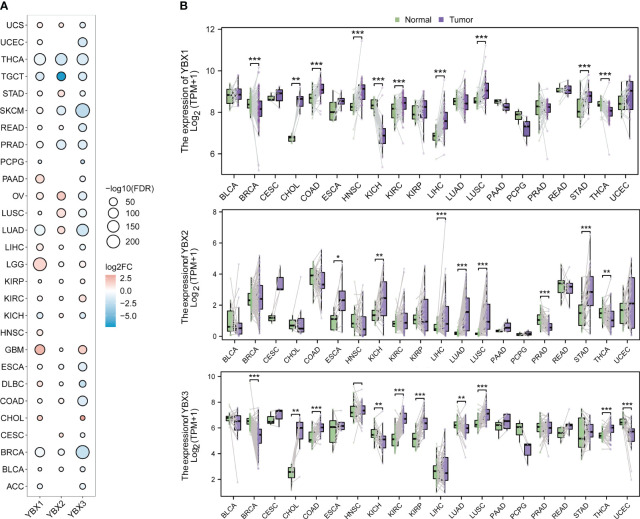
The different mRNA expression of YBX family genes. **(A)** Bubble plot of the different mRNA expression of YBX family between tumor and normal tissues based on the TCGA and the GTEx datasets (FC, fold change; FDR, false discovery rate). **(B)** Violin plot of the different mRNA expression of YBX family in paired tumor and adjacent normal tissues based on the TCGA dataset (*p < 0.05, **p < 0.01, ***p < 0.001).

The expression level of three genes from the YBX family was examined across multiple cancers using the TCGA and GTEx database. **Figure 2A** displays the frequent differential expression of these genes across 28 types of tumors. The expression of three YBX family genes is consistently reduced in thyroid carcinoma (THCA) and testicular germ cell tumors (TGCT), while lower grade glioma (LGG) and glioblastoma multiforme (GBM) consistently show an increased expression. After that, we examined the expression of each YBX family gene in different types of tumors, comparing the tumor tissues with the nearby normal tissues (**Figure 2B**). The pan-cancer analysis discovered that there was a notable increase in the expression of YBXs in tumor tissues, suggesting their potential role as tumor promoters across various types of tumors.

### Prognostic value, single nucleotide variation and copy number variation of YBX family genes

**Figure 3:**
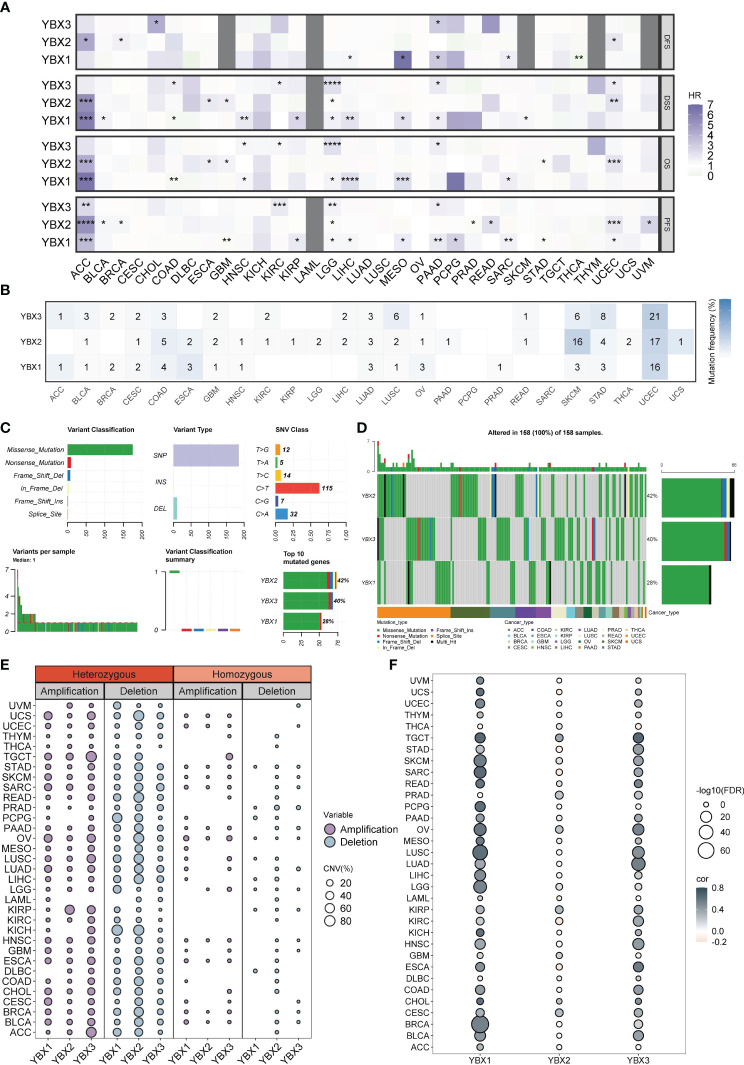
The prognostic value and the SNV alteration of YBX family genes. **(A)** Heatmap of the prognostic value of YBX family in each tumor type. Grey portions indicate values that do not apply. (HR, hazard ratio; *p < 0.05, **p < 0.01, ***p < 0.001, ****p < 0.0001). **(B)** The SNV profile of YBX family in each tumor type. **(C)** The SNV summary of YBX family in pan-cancer. **(D)** Oncoplot of the mutation distribution of YBX family in pan-cancer. **(E)** The heterozygous and homozygous CNV profile of YBX family in each tumor type, including the percentage of amplification and deletion. **(F)** Bubble plot of the correlations between CNV and mRNA expression of YBX family in pan-cancer (FDR, false discovery rate).

We performed a Kaplan-Meier analysis in pan-cancer context for each YBX gene. In numerous types of cancer, YBX emerged as a notable risk factor impacting disease-specific survival (DSS), overall survival (OS), and progression-free survival (PFS). Specifically, YBX1 emerged as significant risk factors for adrenocortical carcinoma (ACC), low LGG, liver hepatocellular carcinoma (LIHC), mesothelioma (MESO). YBX2 served as the determinant for ACC, specifically uterine corpus endometrial carcinoma (UCEC). YBX3 is observed in LGG, PAAD, and KIRC, as shown in **Figure 3A**. On the other hand, YBXs have a defensive function in specific categories of cancers, like YBX1 in colorectal adenocarcinoma (COAD) and THCA, along with YBX2 in stomach adenocarcinoma (STAD). SNV primarily denotes the alteration of an individual nucleotide within a genetic sequence, exerting a pivotal influence on the initiation, advancement, and dissemination of cancerous formations. We additionally investigated the diversity of the YBX gene family variants. **Figures 3B–D** displayed the findings. In UCEC, the YBX family genes exhibit a very high level of SNV mutations, ranging from 16% to 21%, while in COAD, LUAD, LUSC, SKCM, and STAD, there is also a certain level of SNV mutation. In other cancer types, the mutation level of YBX family genes is not high. Multiple kinds of variation were found, among which the missense mutation occurred most frequently. The dominant variant type was SNP, particularly C to T. Aberrant CNV represents a critical molecular mechanism in the development of tumors.

Further analysis revealed that the CNV pattern within the YBX family primarily comprised heterozygous alterations. Notably, the occurrence of heterozygous deletions, particularly in YBX2, was more common than heterozygous amplifications (**Figure 3E**). The analysis of multiple types of cancer revealed a strong association between copy number variation (CNV) and messenger RNA (mRNA) expression. It is worth mentioning that there is a positive correlation between the copy CNV of YBX1 and its mRNA expression level in 30 out of 33 types of tumors (**Figure 3F**).

### The methylation levels of YBX family genes and the correlations between YBXs score and hallmark pathways

**Figure 4:**
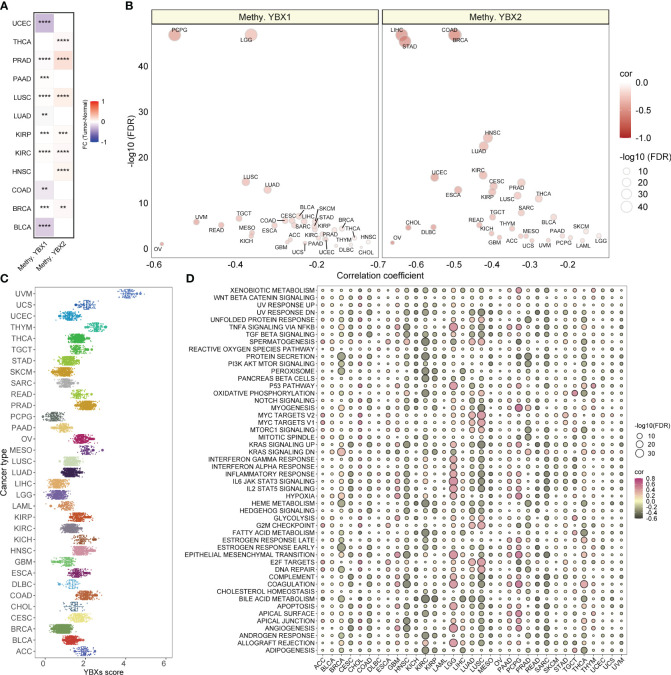
The methylation levels of YBX family genes and the YBXs score distribution and the correlations between YBXs score and hallmark pathways. **(A)** Heatmap of the different methylation levels of YBX family in pan−cancer (FC, fold change; **p < 0.01, ***p < 0.001, ****p < 0.0001). **(B)** Bubble plot of the correlations between the methylation levels and mRNA expression of YBX family in pan−cancer (FDR, false discovery rate). **(C)** The YBXs score distribution in the TCGA pan−cancer cohort. **(D)** Bubble plot of the correlations between YBXs score and hallmark pathways in each tumor type. Pink indicates a positive correlation and black indicates a negative correlation. (FDR, false discovery rate).

The suppression of target genes is facilitated by methylation, an epigenetic modification implicated in tumorigenesis and tumor progression. Our investigation revealed a substantial increase in the methylation level of YBX1 compared to adjacent tissues in the majority of tumors, while the methylation level of YBX2 exhibited a significant decrease (**Figure 4A**). This discovery implied that the abnormal methylation of YBX genes participated in the tumorigenesis and the progression in some type of tumors. Furthermore, it was observed in **Figure 4B** that the YBX family genes exhibited an inverse correlation with the methylation levels of their promoters across various types of cancer, particularly in PCPG, LGG, LIHC, STAD, COAD, and BRCA.

The YBXs score was computed utilizing the ssGSEA algorithm for single-sample gene set enrichment analysis in the TCGA group. The examination showed a fairly even spread of the YBXs score among various types of cancer, with UVM exhibiting the greatest level of expression (**Figure 4C**). Furthermore, the YBXs score was assessed by computing scores for 50 hallmark pathways across pan-cancer pathways. Surprisingly, a notable association was found between the YBXs score and these pathways. Moreover, the correlation between the YBXs score and the scores of the 50 hallmark pathways in each distinct type of cancer was examined (**Figure 4D**). The findings indicated an association between the YBXs score and numerous classic pathways.

### The correlations between YBXs score, TME, and immune checkpoints

**Figure 5:**
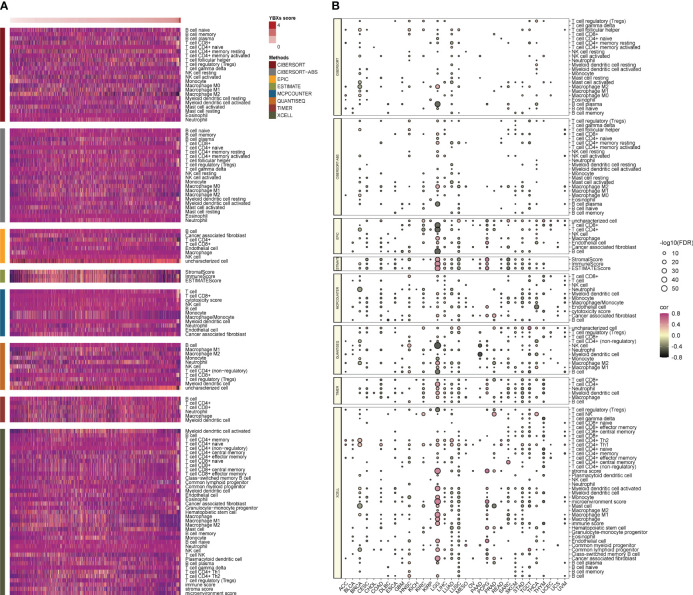
The correlations between YBXs score and tumor microenvironment as well as immune checkpoints. **(A)** Heatmap of the tumor microenvironment scores calculated by different algorithms. **(B)** Heatmap of the correlations between YBXs score and immune checkpoints in each tumor type (FDR, false discovery rate.

A comprehensive analysis was performed to establish a possible correlation between the YBXs score and TME across diverse cancer types. The heatmap clearly indicated an upregulation of most immune cells in conjunction with an escalating YBXs score, a finding corroborated by various algorithms (**Figure 5A**). This pattern was consistently observed across all individual cancer types (**Figure 5B**).

### Examining the associations among the scores of YBXs, stemness, and immunogenicity

**Figure 6:**
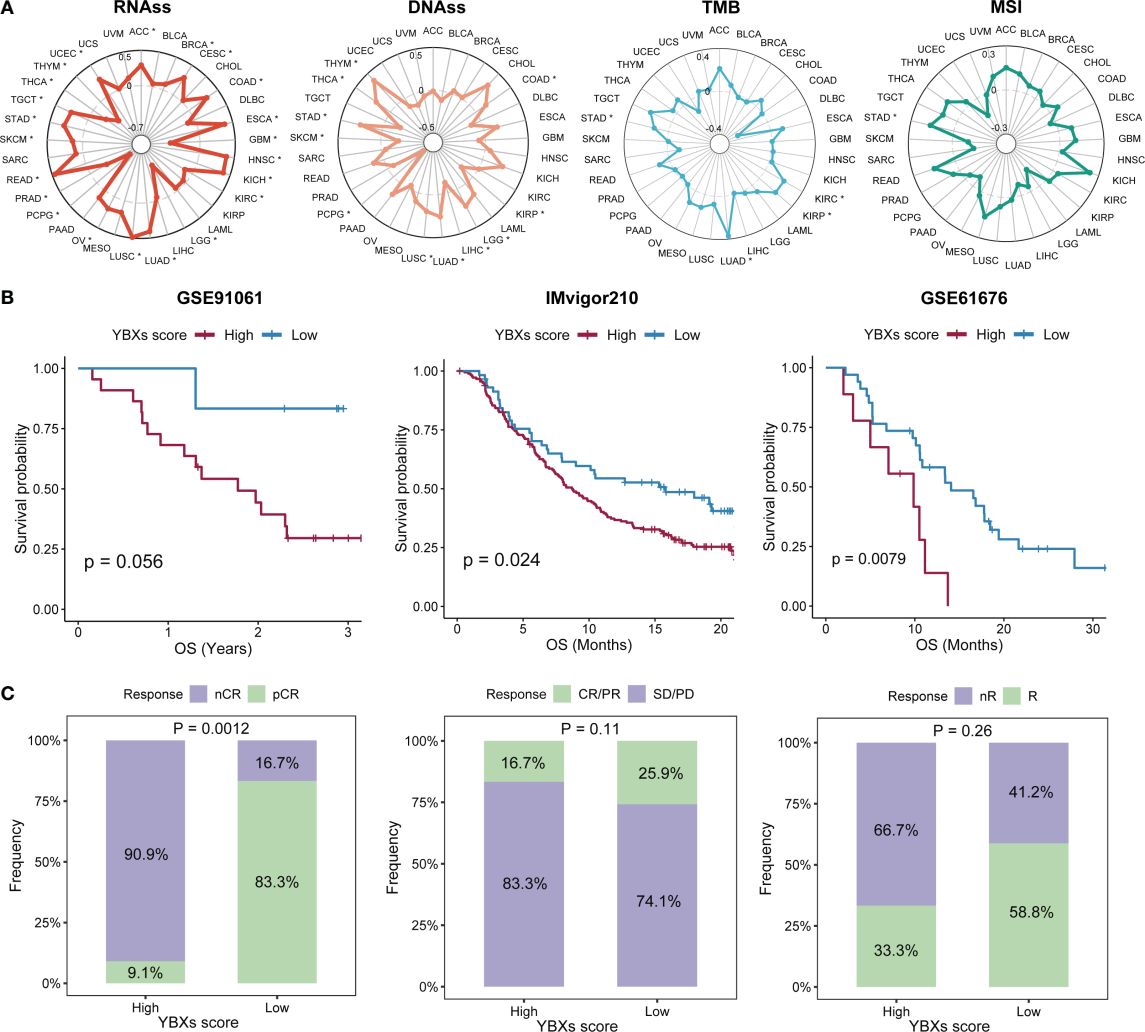
The correlations between YBXs score and stemness and immunogenicity, as well as the predictive efficacy of YBXs score in immunotherapy cohorts. **(A)** The correlations between YBXs score and RNAss, DNAss, TMB, and MSI (*p < 0.05). **(B)** The Kaplan–Meier survival analyses of YBXs score in GSE91061, IMvigor210, and GSE61676 cohorts. **(C)** Results of chi-square test between responsiveness and YBXs groups in GSE91061, IMvigor210, and GSE61676 cohorts. nCR, non-complete response; pCR, pathologic complete response; SD, stable disease; PD, progressive disease.

The examination of stem cell characteristics and immune response demonstrated a correlation with the advancement of tumors. The correlation between the YBXs score and these parameters varied. In most types of cancer, there was generally an observed positive correlation between the score of YBXs and the score of RNA stemness (RNAss). In contrast, other indicators such as DNA stemness score (DNAss) exhibited variability among different cancer types (**Figure 6A**). Nevertheless, only a limited number of cancer types exhibited a notable association between their YBXs score and the levels of mutation burden (TMB) and microsatellite instability (MSI).

### Assessing the prognostic accuracy of YBXs score in diverse cohort types

Initially, we curated several immunotherapy cohorts (GSE91061, IMvigor210, and GSE61676), and computed the YBXs score for each of these datasets. The Kaplan-Meier analysis unveiled that patients undergoing immunotherapy with elevated YBXs scores experienced enhanced OS (**Figure 6B**). Higher YBXs scores associated with better patient responses in all cohorts, suggesting that patients with elevated YBXs scores might derive benefits from immunotherapy (**Figure 6C**).

**Figure 7:**
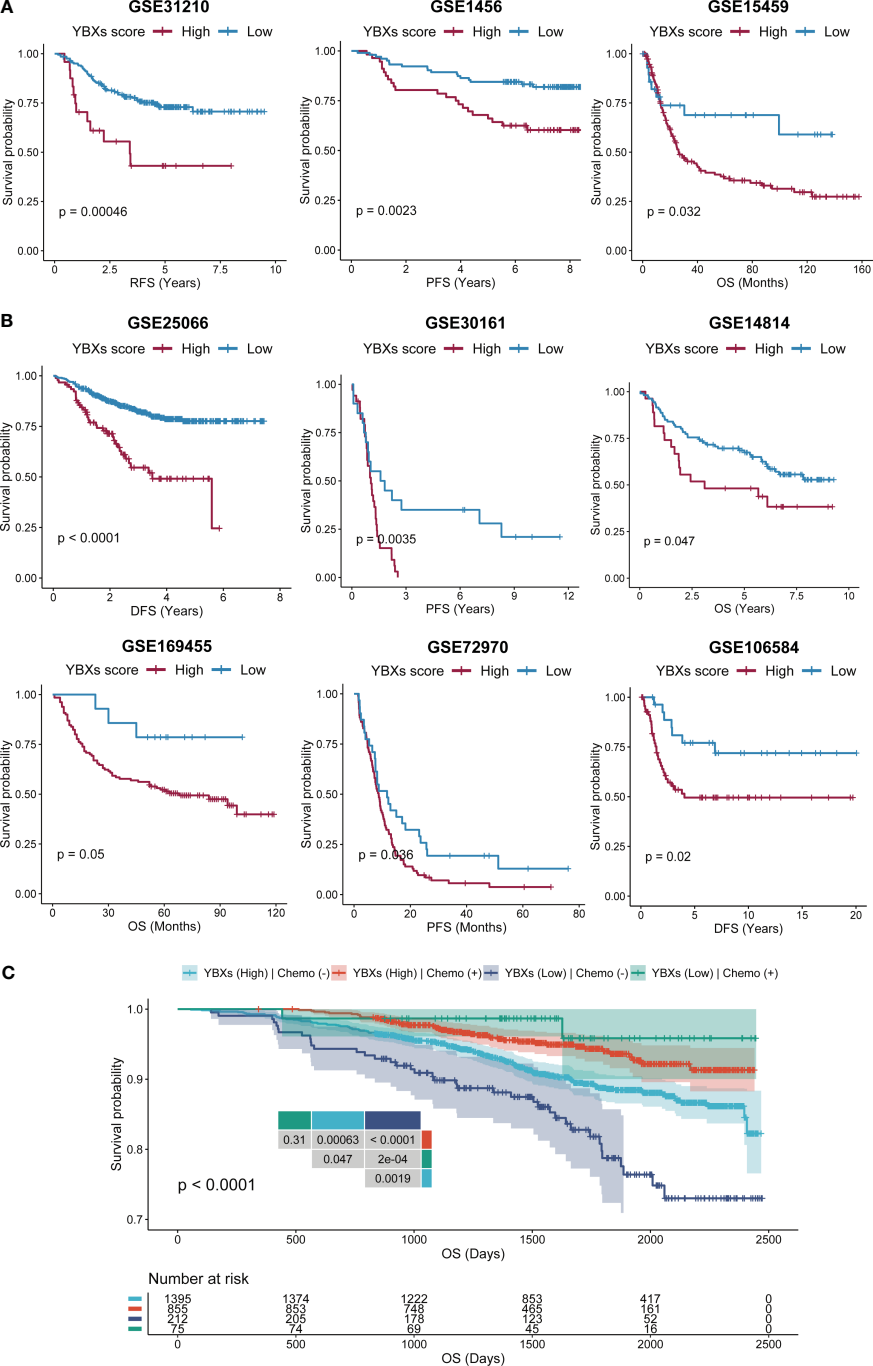
The Kaplan–Meier survival analyses of YBXs score in multi-type of cohorts. **(A)** The Kaplan–Meier survival analyses of YBXs score in post-surgery cohorts. **(B)** The Kaplan–Meier survival analyses of YBXs score in chemotherapy cohorts. **(C)** The Kaplan–Meier survival analyses of YBXs score in GSE96058 cohort.

We expanded our evaluation to investigate whether the YBXs score could predict clinical outcomes in patients who received surgery or chemotherapy across various cohorts. Our analysis indicated that post-surgery patients with low YBXs scores demonstrated better OS, RFS, and PFS (**Figure 7A**). Likewise, individuals undergoing chemotherapy and having low YBXs scores demonstrated improved OS, RFS, PFS, and DFS as depicted in **Figure 7B**. The investigation found that patients who had low YBXs scores and received chemotherapy had the highest OS rates (**Figure 7C**).

### Heatmap of YBXs expression among different cell types in TISCH database

**Figure 8:**
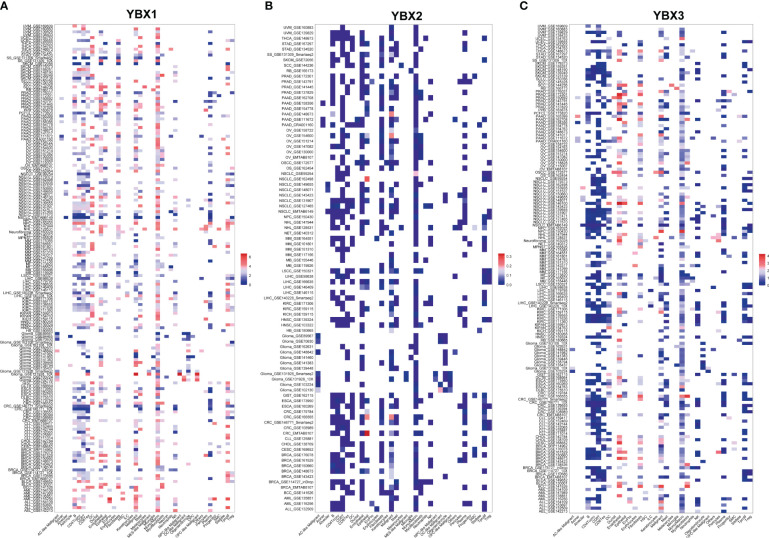
The expression levels of YBX family at single-cell levels. **(A)** The expression levels of YBX1 using TISCH database. **(B)** The expression levels of YBX2 using TISCH database. **(C)** The expression levels of YBX3 using TISCH database.

A further analysis was conducted to determine the YBXs expression among different cell types at single-cell levels in TISCH database (**Figures 8A–C**). Most cohorts revealed elevated expression of both YBX1 and YBX2 in endothelial and malignant cells. Furthermore, YBX1 was observed to be overexpressed in DC cells, NK cells, plasma cells, mono/macro cells, and T proliferating cells. YBX2 exhibited minimal change in most cohorts, except for CRC and NSCLC, where it also showed overexpression in endothelial and malignant cells.

### The knock-down of YBX2 inhibits the cell viability, migration and invasion in HCC cell lines

**Figure 9:**
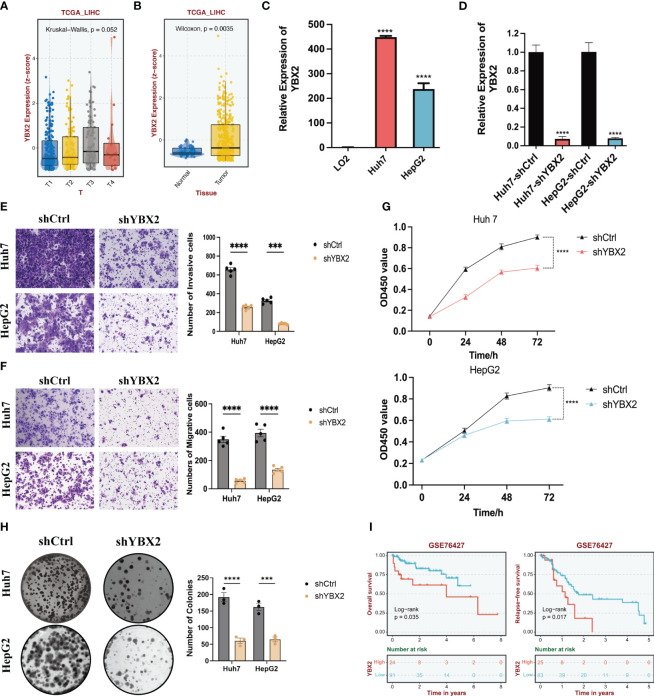
The bioinformatics and experimental validations of YBX2 in HCC cell lines. **(A)** Boxplots showing the expression levels of YBX2 in TCGA-LIHC among different Tumor sizes. **(B)** Boxplots showing the expression levels of YBX2 in TCGA-LIHC between normal and tumor tissues. **(C)** Barplots showing the expression levels of YBX2 in HCC cell lines. **(D)** Barplots showing the expression levels of YBX2 in shCtrl and shYBX2 groups in HCC cell lines. **(E, F)** Transwell assays showing that YBX2 knockdown inhibits the cell migration in HCC cell lines. **(G)** MTT assays showing that YBX2 knockdown inhibits the cell viability in HCC cell lines. **(H)** Colony assays showing that YBX2 knockdown inhibits the cell viability in HCC cell lines. **(I)** The Kaplan–Meier survival analyses of YBX2 in HCC cohorts. ***p < 0.001, ****p < 0.0001.

To further validate the correlation between YBXs expression and tumor behavior, we conducted an analysis using the normal liver tissue and HCC datasets from the TCGA database (**Figures 9A, B**). Although YBX2 expression was below average in both liver tissue and HCC, it exhibited a significantly higher level in HCC compared to liver tissue. Overall, YBX2 showed increased expression in advanced HCC stages. In the HCC cell lines, YBX2 expression levels were observed to rise notably in Huh7 and HepG2 (**Figure 9C**). In order to evaluate the influence of YBX2 on the biological characteristics of HCC cells, we established YBX2-knockdown cell lines and utilized Transwell experiments, MTT tests, and colony formation examinations to assess cell survival, migration, invasion potential, as well as long-term growth and tumor formation effects (**Figure 9D**). Migration and invasion assays were performed (**Figures 9E, F**), showing a significant decrease in the migrative and invasive rates of Huh7 and HepG2 cells. Subsequently, colony formation assays (**Figures 9G, H**) were conducted to investigate the long-term effects of YBX2, revealing a notable reduction in viability and colony-forming ability in YBX2-knockdown cells. Besides, we performed K-M survival analyses using GEO datasets, and we found that higher YBX2 expression was significantly associated with worse prognosis in HCC patients (**Figure 9I**).

## Discussion

The Y-box binding proteins belong to the cold-shock domain protein superfamily, recognized as the most highly conserved family of nucleic acid-binding proteins. Initially, YBX family’s most researched protein, the human YB-1, coded by YBX1, was reported to have a specific binding to the Y-box sequences found in the promoter of the gene for major histocompatibility complex class II (MHC II) (1). The binding of Y-box binding proteins to DNA/RNA helps regulate the transcription and translation processes. The interaction of YB-1 with DNA/RNA plays a pivotal role in regulating both transcription and translation. It has the ability to directly attach to Y-box and similar sequences, either on its own or in combination with other transcription factors (TFs). In addition, YBX1 interacts with different transcription factors, functioning as a collaborator or inhibitor. YBX1 attaches to the unpaired segment of the promoter, either augmenting or impeding the DNA binding of TFs (35). Additionally, YBX1’s binding to mRNA obstructs the cap-binding factor’s access to the mRNA mGpppN cap, thereby repressing translation via a mechanism termed mRNA masking (36).

We extensively analyzed the expression and prognostic importance of four YBX family genes in different types of cancers by utilizing data from the TCGA and GTEx databases. The findings revealed elevated expression levels of YBX family genes in tumor tissues across most cancer types, aligning with the established understanding of YBXs as tumor promoters. The involvement of YBXs in various disease states, including cancer, has been recognized due to dysregulation. Afterwards, we conducted a thorough examination of genetic changes in YBX family genes, including mutations and methylations. The results indicated that the deletion of YBXs genes is associated with tumor progression. It has been demonstrated by numerous studies that YBX1 can act as both oncogenic and tumor-suppressive gene in malignant transformation. A prooncogenic role for YBX1 is suggested by previous studies that the increased expression of YBX1 is associated with cancer aggressiveness in various cancers, including breast carcinoma, colorectal cancer sarcoma and intrahepatic cholangiocarcinoma (6, 12, 18, 37). Moreover, there is significant proof indicating that YBX2 is involved in the maintenance of stem cells and the development of different types of tumors, such as oral and endometrial cancers (22–24). Contrarily, YBX1, when localized in cytoplasm, exhibits tumor suppressive activity through its involvement in the translational silencing of pro-growth transcripts (38, 39). Additionally, our research indicates that the presence of YBXs is linked to a less favorable outlook in the majority of cancer types, although it does provide a safeguarding function in certain forms of cancer. The analysis revealed that YBX1 was found to be a potential risk factor for various types of cancer, such as ACC, LGG, LIHC, and MESO. Likewise, YBX2 is connected to a higher chance of developing ACC and UCEC, whereas YBX3 is associated with LGG, PAAD, and KIRC. Nevertheless, YBXs might have a safeguarding function in specific categories of malignancies, like YBX1 in colon adenocarcinoma (COAD) and thyroid carcinoma (THCA), and YBX2 in stomach adenocarcinoma (STAD). Taken together, these findings indicate that YBX family genes exhibit differential expression across various cancers and possess distinct prognostic significance in different cancer types. Nonetheless, the mechanisms underlying these observations warrant further investigation.

Our study also explored the relationship between YBXs and TME. The tumor microenvironment (TME) plays a vital part in the advancement of tumors and has a strong correlation with the immunotherapy response (40–42). The YBXs score was computed using the ssGSEA method. in the TCGA cohort. Our study demonstrated a relatively consistent distribution of the YBXs score across various cancer types, with UVM exhibiting the highest expression level. We also discovered that the YBXs score exhibited significant correlations with numerous malignant pathways in pan-cancer analysis. A prior investigation validated that YBX1 has the ability to trigger the expression of PD-L1 by attaching to a motif in the PD-L1 promoter, thereby amplifying drug resistance in hepatocellular carcinoma. Moreover, evidence has demonstrated that the nuclear translocation of YBX1 promotes mucin expression, thereby contributing to the survival of pancreatic ductal adenocarcinoma cells within TME (29).

In our investigation, it was observed that patients undergoing immunotherapy with a low YBXs score exhibited diminished OS and PFS. Furthermore, lower YBXs scores were correlated with increased responsiveness among patients in each cohort, suggesting potential benefits of immunotherapy for individuals with a low YBXs score. Moreover, our research revealed that individuals who received surgical intervention or chemotherapy and had a low YBXs score demonstrated enhanced OS, RFS, and PFS in various groups, which is consistent with the results of previous studies (14, 30, 43, 44). We subsequently evaluated the YBXs score’s ability to reflect patient responses across various treatments, finding the results to be promising. Overall, these observations suggest that the YBXs score is closely associated with the TME and may serve as a potential biomarker for predicting the efficacy of diverse treatments, particularly immunotherapy.

Although the relationship between YBX1 and tumor progression has been validated by numerous studies, experimental validation of other genes in the YBX family remains limited. Therefore, we verified the relationship between YBX2 alteration and oncogenic behavior in HCC. Overall, YBX2 exhibited increased expression in late-stage HCC. We observed significantly elevated levels of YBX2 expression in In HCC cell lines. We then established YBX2-knockdown cell lines, and evaluated cell survival, migration, invasion potential, as well as long-term growth and tumor formation effects using Transwell assays, MTT tests, and colony formation assays. Migration and invasion assays were conducted, revealing significantly reduced migration and invasion rates in HCC cell lines. Subsequently, colony formation assays showed significantly decreased survival and colony formation ability in YBX2-knockdown cells.

However, there are some limitations in this study. First, our results are widely based on big-data analyses, which limits our study. Therefore, further experiments using novel technologies, such as spatial transcriptome analyses, are necessary to verify our findings and determine the underlying mechanisms. Second, further verifications are still required to determine how YBXs score can be translated into clinical treatment for patients. Third, prospective cohorts should be included to validate our findings.

## Materials and methods

### Data collection

From the UCSC Xena database, we acquired RNA-sequencing (RNA-seq) profiles that were normalized and converted to log2, along with transcripts per million (TPM) and the corresponding clinical data of the TCGA and GTEx. The “GeoTcgaData” R package was utilized to transform the ensemble ids into gene symbols. Several therapeutic groups were obtained from the Gene Expression Omnibus (GEO) database, including GSE91061, IMvigor210, GSE61676, GSE31210, GSE1456, GSE15459, GSE25066, GSE30161, GSE14814, GSE169455, GSE72970, and GSE106584. Additionally, a previous study called CheckMat (45, 46) was also included. If necessary, the probes were mapped utilizing the ‘AnnoProbe’ R software package. When required, the ‘limma’ R package was utilized to calculate the average of multiple probes (47, 48). We utilized the Gene Set Cancer Analysis (GSCA) database to evaluate changes in genes within the YBX family, encompassing variations such as single nucleotide variants (SNV), copy number variations (CNV), and methylation. For differential methylation, only those have over 10 pairs of tumor-normal samples have been analyzed. Fifty hallmark pathways were acquired from the Molecular Signature Database (MSigDB) and analyzed as described earlier (49). All datasets contained in this study were listed in **Supplementary Tables S1**, **S2**.

### YBXs score and tumor microenvironment analysis

Using the ssGSEA algorithm (‘GSVA’ R package) (50), we computed the YBXs score for each patient. R package ‘immunedeconv’ (51) was used to calculate the infiltration data of immune cells from the TCGA cohort.

### Clinical outcome analysis

A K–M analysis was performed by using the “survival” and “survminer” R packages to determine if the YBXs score correlates with survival outcomes (OS, DSS, DFS, and PFS). The optimal cut-off point was determined using the function ‘surv_cutpoint’. ROC curve analysis was conducted using the R package ‘pROC’ (52). To forecast the effectiveness of responsiveness, we utilized both a Chi-square test and Fisher’s exact test.

### Cell culture and transfection

The American type culture collection was the source of the purchased cell lines. HepG2, Huh7, and LO2 cells were grown in DMEM (Gibco, USA) containing 10% fetal bovine serum (Gibco, USA) and antibiotics at a temperature of 37°C with 5% CO2. HCC cell lines were transfected with shRNAs targeting YBX2 or control using (**Supplementary Table S3**). The effectiveness of the knockdown was verified through qRT-PCR examination.

### Cell proliferation experiment

Cell proliferation experiments were conducted using the CCK-8 kit obtained from MedChemExpress, USA. At first, the cells were placed in 96-well dishes with a concentration of 4x103 cells/mL and kept at a temperature of 37°C. After specific time periods (0, 24, 48, and 72 hours) had elapsed, 10 μL of CCK-8 solution was introduced into each well, followed by continued incubation of the cells at 37°C. At last, the spectrophotometer was used to measure the absorbance of samples that had undergone various treatments at a wavelength of 450 nm.

### Quantitative real time polymerase chain reaction

The One Step TB Green® PrimeScript™ PLUS RT-PCR Kit (Takara, Beijing, China) was used to isolate total RNA from HepG2, Huh7, and LO2 cell lines, following the manufacturer’s protocol. Gene expression in HCC cell lines was measured using real-time PCR with SYBR-Green-based (Applied Biosystems® SYBR® Green PCR Master Mix) on the StepOnePlus™ System (Thermo Fisher Scientific Life Sciences). The 2−ΔΔCT method was employed to normalize the expression level of target genes to GAPDH, which served as a reference gene. The primers sequences were involved in **Supplementary Table S4**.

### Transwell assay

In the invasion assay, only matrigel (BD Biosciences) and fibronectin were employed for transwell migration and invasion assays. Polymerization of 10% matrigel was carried out at the bottom of the upper chamber of a 24 well transwell plate for 30 minutes at a temperature of 37°C. After being deprived of serum for 16 hours, the aforementioned HCC cells were introduced into the upper chambers. HepG2 had a cell concentration of 3x10 (5) per well, while Huh-7 had a cell concentration of 1x10 (6) per well. In the migration experiment, the incubation time for the cells mentioned above was 24 hours. In the invasion experiment, the incubation time for these cells was 48 hours. DMEM was filled with 20% fetal bovine serum in the lower chambers. After incubation, we used the cotton swabs to remove the non-invading cells from the upper surface of the membrane. The invading or migrating cells were immobilized in 4% paraformaldehyde for 30 minutes and subsequently treated with 0.1% crystal violet for 30 minutes at ambient temperature. Water was used to remove the surplus dye.

### Colony formation assay

The HCC cells with control and YBX2 knockdown were separately placed in six-well plates with a density of 500 cells per well and were incubated at 37°C in a 5% CO2 humid atmosphere. Following a 2-week incubation period, the plates were rinsed with PBS and subsequently treated with methanol for 1 hour. They were then stained with crystal violet for a duration of 30 minutes. Afterward, the plates were rinsed with fresh water and the quantity of the aforementioned cell colonies was tallied.

### Statistical analysis

R 4.1.0 was used to present all the statistical analyses. To examine the disparities between the two groups, the Student’s t-test was employed. Survival curves were plotted using K–M plots and compared using log-rank tests. Pearson correlations were used to calculate the correlation coefficients. The Benjamini and Hochberg method was used to calculate the rate of false discovery. Statistical significance was assessed based on p-values below 0.05.

## Conclusion

The results of this investigation elucidate the pivotal role of YBX family genes in both tumor progression and tumorigenesis. The YBXs score demonstrates a strong correlation with the TME and shows potential as a biomarker for predicting the efficacy of diverse treatments. The research provides valuable knowledge about the possible anti-cancer mechanisms controlled by YBX family genes, which justifies the need for additional comprehensive investigation and confirmation.

## Data availability statement

The datasets presented in this study can be found in online repositories. The names of the repository/repositories and accession number(s) can be found in the article/**Supplementary Material**.

## Ethics statement

Ethical approval was not required for the studies on animals in accordance with the local legislation and institutional requirements because only commercially available established cell lines were used.

## Author contributions

ZY: Writing – original draft, Formal analysis, Data curation. BL: Data curation, Formal analysis, Writing – original draft. WL: Data curation, Formal analysis, Writing – original draft. DK: Writing – original draft, Formal analysis. XD: Writing – original draft, Validation. HT: Writing – original draft, Validation. JX: Conceptualization, Writing – review & editing. DH: Conceptualization, Funding acquisition, Writing – review & editing. AC: Conceptualization, Writing – review & editing.

## References

[1] Didier DK Schiffenbauer J Woulfe SL Zacheis M Schwartz BD. Characterization of the cDNA encoding a protein binding to the major histocompatibility complex class II Y box. Proc Natl Acad Sci USA. (1988) 85:7322–6. doi: 10.1073/pnas.85.19.7322 PMC282178 3174636

[2] Tafuri SR Wolffe AP. Xenopus Y-box transcription factors: molecular cloning, functional analysis and developmental regulation. Proc Natl Acad Sci USA. (1990) 87:9028–32. doi: 10.1073/pnas.87.22.9028 PMC55094 2247479

[3] Kohno K Izumi H Uchiumi T Ashizuka M Kuwano M. The pleiotropic functions of the Y-box-binding protein, YB-1. BioEssays. (2003) 25:691–8. doi: 10.1002/bies.10300 12815724

[4] Kohno Y Matsuki Y Tanimoto A Izumi H Uchiumi T Kohno K. Expression of Y-box-binding protein dbpC/contrin, a potentially new cancer/testis antigen. Br J Cancer. (2006) 94:710–6. doi: 10.1038/sj.bjc.6602987 PMC2361212 16479255

[5] Zhang J Fan JS Li S Yang Y Sun P Zhu Q. Structural basis of DNA binding to human YB-1 cold shock domain regulated by phosphorylation. Nucleic Acids Res. (2020) 48(16):9361–71. doi: 10.1093/nar/gkaa619 PMC7498358 32710623

[6] Ladomery M Sommerville J. A role for Y-box proteins in cell proliferation. Bioessays. (1995) 17:9–11. doi: 10.1002/bies.950170104 7702598

[7] Wu SL Fu X Huang J Jia TT Zong FY Mu SR. Genome-wide analysis of YB-1-RNA interactions reveals a novel role of YB-1 in miRNA processing in glioblastoma multiforme. Nucleic Acids Res. (2015) 43(17):8516–28. doi: 10.1093/nar/gkv779 PMC4787835 26240386

[8] Jayavelu AK Schnöder TM Perner F Herzog C Meiler A Krishnamoorthy G. Splicing factor YBX1 mediates persistence of JAK2-mutated neoplasms. Nature. (2020) 588(7836):157–63. doi: 10.1038/s41586-020-2968-3 33239784

[9] Evdokimova V Tognon C Ng T Ruzanov P Melnyk N Fink D. Translational activation of snail1 and other developmentally regulated transcription factors by YB-1 promotes an epithelial-mesenchymal transition. Cancer Cell. (2009) 15(5):402–15. doi: 10.1016/j.ccr.2009.03.017 19411069

[10] Mouneimne G Brugge JS. YB-1 translational control of epithelial-mesenchyme transition. Cancer Cell. (2009) 15:357–9. doi: 10.1016/j.ccr.2009.04.006 19411064

[11] Goodarzi H Liu X Nguyen HCB Zhang S Fish L Tavazoie SF. Endogenous tRNA-Derived Fragments Suppress Breast Cancer Progression via YBX1 Displacement. Cell. (2015) 161:790–802. doi: 10.1016/j.cell.2015.02.053 25957686 PMC4457382

[12] El-Naggar AM Veinotte CJ Cheng H Grunewald TGP Negri GL Somasekharan SP. Translational activation of HIF1α by YB-1 promotes sarcoma metastasis. Cancer Cell. (2015) 27(5):682–97. doi: 10.1016/j.ccell.2015.04.003 25965573

[13] Tao Z Ruan H Sun L Kuang D Song Y Wang Q. Targeting the YB-1/PD-L1 axis to enhance chemotherapy and antitumor immunity. Cancer Immunol Res. (2019) 7(7):1135–47. doi: 10.1158/2326-6066.CIR-18-0648 31113805

[14] Schelch K Emminger D Zitta B Johnson TG Kopatz V Eder S. Targeting YB-1 via entinostat enhances cisplatin sensitivity of pleural mesothelioma *in vitro* and *in vivo* . Cancer Lett. (2023) 574:216395. doi: 10.1016/j.canlet.2023.216395 37730104

[15] Bai Y Gotz C Chincarini G Zhao Z Slaney C Boath J. YBX1 integration of oncogenic PI3K/mTOR signalling regulates the fitness of Malignant epithelial cells. Nat Commun. (2023) 14(1):1591. doi: 10.1038/s41467-023-37161-0 36949044 PMC10033729

[16] Xu J Ji L Liang Y Wan Z Zheng W Song X. CircRNA-SORE mediates sorafenib resistance in hepatocellular carcinoma by stabilizing YBX1. Signal Transduct Target Ther. (2020) 5(1):298. doi: 10.1038/s41392-020-00375-5 33361760 PMC7762756

[17] Du M Hu X Jiang X Yin L Chen J Wen J. LncRNA EPB41L4A-AS2 represses Nasopharyngeal Carcinoma Metastasis by binding to YBX1 in the Nucleus and Sponging MiR-107 in the Cytoplasm. Int J Biol Sci. (2021) 17(8):1963–78. doi: 10.7150/ijbs.55557 PMC8193272 34131399

[18] Chen Q Wang H Li Z Li F Liang L Zou Y. Circular RNA ACTN4 promotes intrahepatic cholangiocarcinoma progression by recruiting YBX1 to initiate FZD7 transcription. J Hepatol. (2022) 76(1):135–47. doi: 10.1016/j.jhep.2021.08.027 34509526

[19] Wang Y Feng YC Gan Y Teng L Wang L La T. LncRNA MILIP links YBX1 to translational activation of Snai1 and promotes metastasis in clear cell renal cell carcinoma. J Exp Clin Cancer Res. (2022) 41(1):260. doi: 10.1186/s13046-022-02452-9 36028903 PMC9414127

[20] Liu B Shen H He J Jin B Tian Y Li W. Cytoskeleton remodeling mediated by circRNA-YBX1 phase separation suppresses the metastasis of liver cancer. Proc Natl Acad Sci USA. (2023) 120(30):e2220296120. doi: 10.1073/pnas.2220296120 37459535 PMC10372620

[21] Yu T Zhang Q Yu SK Nie FQ Zhang ML Wang Q. THOC3 interacts with YBX1 to promote lung squamous cell carcinoma progression through PFKFB4 mRNA modification. Cell Death Dis. (2023) 14(7):475. doi: 10.1038/s41419-023-06008-3 37500615 PMC10374565

[22] Cai Y Li N Li H. YBX2 modulates mRNA stability via interaction with YTHDF2 in endometrial cancer cells. Exp Cell Res. (2023) 427(1):113586. doi: 10.1016/j.yexcr.2023.113586 37030331

[23] Suzuki I Yoshida S Tabu K Kusunoki S Matsumura Y Izumi H. YBX2 and cancer testis antigen 45 contribute to stemness, chemoresistance and a high degree of Malignancy in human endometrial cancer. Sci Rep. (2021) 11(1):4220. doi: 10.1038/s41598-021-83200-5 33602962 PMC7893073

[24] Guo L Lin Q Zhao X Xu J. Circular CDC like kinase 1 suppresses cell apoptosis through miR-18b-5p/Y-box protein 2 axis in oral squamous cell carcinoma. Bioengineered. (2022) 13:4226–34. doi: 10.1080/21655979.2022.2027174 PMC8973868 35156507

[25] Chen F Liu M Yu Y Sun Y Li J Hu W. LINC00958 regulated miR-627-5p/YBX2 axis to facilitate cell proliferation and migration in oral squamous cell carcinoma. Cancer Biol Ther. (2019) 20(9):1270–80. doi: 10.1080/15384047.2019.1617571 PMC6741574 31161900

[26] Ma M Chen Y Chong X Jiang F Gao J Shen L. Integrative analysis of genomic, epigenomic and transcriptomic data identified molecular subtypes of esophageal carcinoma. Aging (Albany NY). (2021) 13(5):6999–7019. doi: 10.18632/aging.202556 33638948 PMC7993659

[27] Sun Y Li Z Wang W Zhang X Li W Du G. Identification and verification of YBX3 and its regulatory gene HEIH as an oncogenic system: A multidimensional analysis in colon cancer. Front Immunol. (2022) 13:957865. doi: 10.3389/fimmu.2022.957865 36059530 PMC9433931

[28] Xie J Zhang H Wang K Ni J Ma X Khoury CJ. M6A-mediated-upregulation of lncRNA BLACAT3 promotes bladder cancer angiogenesis and hematogenous metastasis through YBX3 nuclear shuttling and enhancing NCF2 transcription. Oncogene. (2023) 42(40):2956–70. doi: 10.1038/s41388-023-02814-3 PMC10541332 37612524

[29] Xie J Deng X Xie Y Zhu H Liu P Deng W. Multi-omics analysis of disulfidptosis regulators and therapeutic potential reveals glycogen synthase 1 as a disulfidptosis triggering target for triple-negative breast cancer. MedComm. (2020) 5:e502. doi: 10.1002/mco2.502 PMC10901283 38420162

[30] Wu Z Wang Y Yan M Liang Q Li B Hou G. Comprehensive analysis of the endoplasmic reticulum stress-related long non-coding RNA in bladder cancer. Front Oncol. (2022) 12:951631. doi: 10.3389/fonc.2022.951631 35992824 PMC9386564

[31] Hu Y Zhang X Li Q Zhou Q Fang D Lu Y. An immune and epigenetics-related scoring model and drug candidate prediction for hepatic carcinogenesis via dynamic network biomarker analysis and connectivity mapping. Comput Struct Biotechnol J. (2023) 21:4619–33. doi: 10.1016/j.csbj.2023.09.030 PMC10561057 37817777

[32] Kanterman J Sade-Feldman M Biton M Ish-Shalom E Lasry A Goldshtein A. Adverse immunoregulatory effects of 5FU and CPT11 chemotherapy on myeloid-derived suppressor cells and colorectal cancer outcomes. Cancer Res. (2014) 74:6022–35. doi: 10.1158/0008-5472.CAN-14-0657 25209187

[33] Daly RJ Scott AM Klein O Ernst M. Enhancing therapeutic anti-cancer responses by combining immune checkpoint and tyrosine kinase inhibition. Mol Cancer. (2022) 21:189. doi: 10.1186/s12943-022-01656-z 36175961 PMC9523960

[34] Oba T Long MD Keler T Marsh HC Minderman H Abrams SI. Overcoming primary and acquired resistance to anti-PD-L1 therapy by induction and activation of tumor-residing cDC1s. Nat Commun. (2020) 11(1):5415. doi: 10.1038/s41467-020-19192-z 33110069 PMC7592056

[35] Wilusz CJ Wormington M Peltz SW. The cap-to-tail guide to mRNA turnover. Nat Rev Mol Cell Biol. (2001) 2:237–46. doi: 10.1038/35067025 11283721

[36] Evdokimova V Ruzanov P Imataka H Raught B Svitkin Y Ovchinnikov LP. The major mRNA-associated protein YB-1 is a potent 5’ cap-dependent mRNA stabilizer. EMBO J. (2001) 20(19):5491–502. doi: 10.1093/emboj/20.19.5491 PMC125650 11574481

[37] Nagasu S Sudo T Kinugasa T Yomoda T Fujiyoshi K Shigaki T. Y−box−binding protein 1 inhibits apoptosis and upregulates EGFR in colon cancer. Oncol Rep. (2019) 41(5):2889–96. doi: 10.3892/or.2019.7038 30864697

[38] Bader AG Vogt PK. Phosphorylation by Akt disables the anti-oncogenic activity of YB-1. Oncogene. (2008) 27:1179–82. doi: 10.1038/sj.onc.1210719 17704806

[39] Bader AG Vogt PK. Inhibition of protein synthesis by Y box-binding protein 1 blocks oncogenic cell transformation. Mol Cell Biol. (2005) 25:2095–106. doi: 10.1128/MCB.25.6.2095-2106.2005 PMC1061623 15743808

[40] Ruf B Heinrich B Greten TF. Immunobiology and immunotherapy of HCC: spotlight on innate and innate-like immune cells. Cell Mol Immunol. (2021) 18:112–27. doi: 10.1038/s41423-020-00572-w PMC7852696 33235387

[41] Lu C Rong D Zhang B Zheng W Wang X Chen Z. Current perspectives on the immunosuppressive tumor microenvironment in hepatocellular carcinoma: challenges and opportunities. Mol Cancer. (2019) 18(1):130. doi: 10.1186/s12943-019-1047-6 31464625 PMC6714090

[42] Prieto J Melero I Sangro B. Immunological landscape and immunotherapy of hepatocellular carcinoma. Nat Rev Gastroenterol Hepatol. (2015) 12:681–700. doi: 10.1038/nrgastro.2015.173 26484443

[43] Wang J Shen D Li S Li Q Zuo Q Lu J. LINC00665 activating Wnt3a/β-catenin signaling by bond with YBX1 promotes gastric cancer proliferation and metastasis. Cancer Gene Ther. (2023) 30(11):1530–42. doi: 10.1038/s41417-023-00657-4 37563362

[44] Ru J Lu J Ge J Ding B Su R Jiang Y. IRGM is a novel regulator of PD-L1 via promoting S6K1-mediated phosphorylation of YBX1 in hepatocellular carcinoma. Cancer Lett. (2023) 581:216495. doi: 10.1016/j.canlet.2023.216495 37993085

[45] Braun DA Hou Y Bakouny Z Ficial M Sant’ Angelo M Forman J. Interplay of somatic alterations and immune infiltration modulates response to PD-1 blockade in advanced clear cell renal cell carcinoma. Nat Med. (2020) 26(6):909–18. doi: 10.1038/s41591-020-0839-y PMC7499153 32472114

[46] Zou Y Xie J Zheng S Liu W Tang Y Tian W. Leveraging diverse cell-death patterns to predict the prognosis and drug sensitivity of triple-negative breast cancer patients after surgery. Int J Surg. (2022) 107:106936. doi: 10.1016/j.ijsu.2022.106936 36341760

[47] Ritchie ME Phipson B Wu D Hu Y Law CW Shi W. limma powers differential expression analyses for RNA-sequencing and microarray studies. Nucleic Acids Res. (2015) 43(7):e47. doi: 10.1093/nar/gkv007 25605792 PMC4402510

[48] Liberzon A Birger C Thorvaldsdóttir H Ghandi M Mesirov JP Tamayo P. The Molecular Signatures Database (MSigDB) hallmark gene set collection. Cell Syst. (2015) 1:417–25. doi: 10.1016/j.cels.2015.12.004 PMC4707969 26771021

[49] Yang L Li N Xue Z Liu LR Li J Huang X. Synergistic therapeutic effect of combined PDGFR and SGK1 inhibition in metastasis-initiating cells of breast cancer. Cell Death Differ. (2020) 27:2066–80. doi: 10.1038/s41418-019-0485-4 PMC7308369 31969692

[50] Hänzelmann S Castelo R Guinney J. GSVA: gene set variation analysis for microarray and RNA-seq data. BMC Bioinf. (2013) 14:7. doi: 10.1186/1471-2105-14-7 PMC3618321 23323831

[51] Sturm G Finotello F Petitprez F Zhang JD Baumbach J Fridman WH. Comprehensive evaluation of transcriptome-based cell-type quantification methods for immuno-oncology. Bioinformatics. (2019) 35:i436–45. doi: 10.1093/bioinformatics/btz363 PMC6612828 31510660

[52] Robin X Turck N Hainard A Tiberti N Lisacek F Sanchez JC. pROC: an open-source package for R and S+ to analyze and compare ROC curves. BMC Bioinf. (2011) 12:77. doi: 10.1186/1471-2105-12-77 PMC3068975 21414208

