# Supplementary material for: Comprehensive pan-cancer analysis of YBX family reveals YBX2 as a potential biomarker in liver cancer
Source: Front Immunol. 2024 Apr 18;15:1382520. doi: 10.3389/fimmu.2024.1382520 (PMC11063299; doi:10.3389/fimmu.2024.1382520)
Supplement: Supplementary file 2 [file Table_2.docx]

**Supplementary Table S1. Summary of TCGA and GTEx sample sizes of different tumor types in this study.**

Abbreviations: ACC, Adrenocortical Cancer; BLCA, Bladder Cancer; BRCA, Breast Cancer; CESC, Cervical Cancer; CHOL, Bile Duct Cancer; COAD, Colon Adenocarcinoma; DLBC, Large B-cell Lymphoma; ESCA, Esophageal Cancer; GBM, Glioblastoma; HNSC, Head and Neck Squamous Cell Carcinoma; KICH, Kidney Chromophobe; KIRC, Kidney Renal Clear Cell Carcinoma; KIRP, Kidney Renal Papillary Cell Carcinoma; LAML, Acute Myeloid Leukemia; LGG, Lower Grade Glioma; LIHC, Liver Hepatocellular Carcinoma; LUAD, Lung Adenocarcinoma; LUSC, Lung Squamous Cell Carcinoma; MESO, Mesothelioma; OV, Ovarian Cancer; PAAD, Pancreatic Cancer; PCPG, Pheochromocytoma & Paraganglioma; PRAD, Prostate Adenocarcinoma; READ, Rectum Adenocarcinoma; SARC, Sarcoma; SKCM, Skin Cutaneous Melanoma; STAD, Stomach Adenocarcinoma; TGCT, Testicular Cancer; THCA, Thyroid Cancer; THYM, Thymoma; UCEC, Uterine Corpus Endometrial Carcinoma; UCS, Uterine Carcinosarcoma; UVM, Ocular melanomas.

| Tumor type | TCGA (Tumor) | TCGA (Normal) | GTEx (Normal) |
| --- | --- | --- | --- |
| ACC | 79 | 0 | 258 |
| BLCA | 406 | 19 | 21 |
| BRCA | 1101 | 113 | 459 |
| CESC | 306 | 3 | 19 |
| CHOL | 35 | 9 | 0 |
| COAD | 455 | 41 | 779 |
| DLBC | 48 | 0 | 929 |
| ESCA | 163 | 11 | 1445 |
| GBM | 153 | 5 | 2642 |
| HNSC | 504 | 44 | 0 |
| KICH | 65 | 25 | 89 |
| KIRC | 532 | 72 | 89 |
| KIRP | 290 | 32 | 89 |
| LAML | 150 | 0 | 0 |
| LGG | 513 | 0 | 2642 |
| LIHC | 371 | 50 | 226 |
| LUAD | 516 | 59 | 578 |
| LUSC | 501 | 49 | 578 |
| MESO | 87 | 0 | 0 |
| OV | 376 | 0 | 180 |
| PAAD | 179 | 4 | 328 |
| PCPG | 181 | 3 | 0 |
| PRAD | 498 | 52 | 245 |
| READ | 165 | 10 | 779 |
| SARC | 260 | 2 | 0 |
| SKCM | 471 | 1 | 1809 |
| STAD | 375 | 32 | 359 |
| TGCT | 134 | 0 | 361 |
| THCA | 512 | 59 | 653 |
| THYM | 120 | 2 | 0 |
| UCEC | 545 | 35 | 142 |
| UCS | 57 | 0 | 142 |
| UVM | 80 | 0 | 0 |

**Supplementary Table S2.** **Datasets enrolled in this study.**

| Dataset | Source |
| --- | --- |
| TCGA-Pan cancer | https://xenabrowser.net/datapages/ |
| GTEx | https://xenabrowser.net/datapages/ |
| GSE91061 | https://www.ncbi.nlm.nih.gov/geo/query/acc.cgi?acc=GSE91061 |
| IMvigor210 | http://research-pub.gene.com/IMvigor210CoreBiologies/IMvigor210CoreBiologies.tar.gz. |
| GSE61676 | https://www.ncbi.nlm.nih.gov/geo/query/acc.cgi?acc=GSE61676 |
| GSE31210 | https://www.ncbi.nlm.nih.gov/geo/query/acc.cgi?acc=GSE31210 |
| GSE1456 | https://www.ncbi.nlm.nih.gov/geo/query/acc.cgi?acc=GSE1456 |
| GSE15459 | https://www.ncbi.nlm.nih.gov/geo/query/acc.cgi?acc=GSE15459 |
| GSE25066 | https://www.ncbi.nlm.nih.gov/geo/query/acc.cgi?acc=GSE25066 |
| GSE30161 | https://www.ncbi.nlm.nih.gov/geo/query/acc.cgi?acc=GSE30161 |
| GSE14814 | https://www.ncbi.nlm.nih.gov/geo/query/acc.cgi?acc=GSE14814 |
| GSE169455 | https://www.ncbi.nlm.nih.gov/geo/query/acc.cgi?acc=GSE169455 |
| GSE72970 | https://www.ncbi.nlm.nih.gov/geo/query/acc.cgi?acc=GSE72970 |
| GSE106584 | https://www.ncbi.nlm.nih.gov/geo/query/acc.cgi?acc=GSE106584 |
| CheckMat | doi:10. 1038/s41591-020-0839-y |

**Supplementary Table S3. shRNAs sequence.**

| Non-targeting | shControl | CTGTCACCACAGTAGCTTGG |
| --- | --- | --- |
| YBX2 | shYBX2 | CCCAACCAGCAGCAGCCTATA |

**Supplementary Table S4. Primers for qRT-PCR detection.**

| YBX2 | Forward | GCTGGCAATCCAAGTCCTG |
| --- | --- | --- |
|  | Reverse | TCAAATTCCACAGTCTCCCCAT |
| GAPDH | Forward | CTGGGCTACACTGAGCACC |
|  | Reverse | AAGTGGTCGTTGAGGGCAATG |
